# Latrepirdine (Dimebon), a potent 5-HT_7_ receptor antagonist, modulates stress-mediated behaviors in rodents

**DOI:** 10.3389/fphar.2026.1771657

**Published:** 2026-05-13

**Authors:** Dinesh K. Dhull, Subhendu Seth, Frank D. Yocca, Michael De Vivo

**Affiliations:** 1 E. Z. BioXcel Solutions Pvt. Ltd., Gurugram, Haryana, India; 2 BioXcel Therapeutics Inc., New Haven, CT, United States

**Keywords:** 5-HT7 receptor antagonist, aggression, agitation, latrepirdine, stress

## Abstract

**Objectives:**

First, determine the likely molecular target of latrepirdine. Second, using stress-mediated rodent behavioral models, determine which clinical neuropsychiatric symptoms are possibly improved by latrepirdine. Third, establish whether latrepirdine possesses properties suitable for chronic treatment of neuropsychiatric symptoms related to stress.

**Methods:**

Characterization of the potency of latrepirdine was performed by both displacement binding with labeled LSD and inhibition of stimulated adenylyl cyclase activity using membranes from human 5-HT_7_ cDNA receptor-transfected cells. *Microdialysis:* Samples of interstitial fluid from brain was collected using microdialysis probes and blood was obtained through a jugular cannulation. *Behavioral models:* Stress-related models included the elevated plus maze with either a yohimbine or CCK-4 challenge, the resident intruder aggression task, open field test, marble burying, latency to REM sleep, forced swim test and the open space swim test.

**Results:**

Binding and adenylyl cyclase activity aligned and indicated that affinity of latrepirdine for 5-HT_7_ receptors was approximately 10 nM. Free brain levels of latrepirdine at effective doses in all models were consistently at the 10 nM level. Latrepirdine was effective after repeat dosing, reversible after stopping dosing, and demonstrated good brain penetration.

**Conclusion:**

Latrepirdine, possibly by blocking 5-HT_7_ receptors in the locus coeruleus, regulates anxiety, aggressive and compulsive behaviors in rodents. These effects may account for the neuropsychiatric effects detected in the clinic. Latrepirdine has properties that suggest it would be a useful drug for the chronic treatment of stress-induced and anxiety-related symptoms.

## Highlights

- Latrepirdine (Dimebon), was in clinical development for treatment of cognition for patients with Alzheimer’s disease but missed its primary endpoint. However, it did improve neuropsychiatric symptoms as measured by the neuropsychiatric inventory scale.
- In this study, latrepirdine was effective, at doses likely to block 5-HT_7_ receptors in brain, in stress-mediated rodent models related to the clinical endpoints measured by the neuropsychiatric inventory.
- In this study, latrepirdine exhibited properties such as good brain penetration, continued efficacy after repeated dosing, and reversibility, that make it potentially useful for chronic treatment of stress-related neuropsychiatric symptoms.

## 1 Introduction

Latrepirdine, also known as Dimebon, was used for many years in Russia as an antihistamine for allergy treatment. Small open-label studies suggested that latrepirdine might be beneficial with respect to cognition and neuropsychiatric symptoms, such as anxiety and depression, in Alzheimer’s patients (Bachurin et al., 2003). As a result of these reports, a clinical study with latrepirdine was conducted in Russia in patients with mild to moderate Alzheimer’s disease. The results of this initial study were positive (Doody et al., 2008). However, in 2 subsequent studies, latrepirdine did not meet its primary endpoints related to cognition (Jones, 2010). Although no benefit on cognition was noted, a pooled analysis from all 3 studies indicated that latrepirdine had a significant effect on the neuropsychiatric inventory (NPI) (Cano-Cuenca et al., 2014; Chau et al., 2015). The NPI is a clinical scale that measures changes in frequency and severity of neuropsychiatric symptoms in Alzheimer’s patients (Cummings, 2020). Domains measured by NPI include anxiety, agitation, irritability and depression. It is not known which domains were responsible for the NPI signal. In addition to this potential efficacy signal, the clinical development program exposed hundreds of Alzheimer’s patients to latrepirdine for up to a year. The data from those studies suggests that latrepirdine was well tolerated and safe.

Lack of understanding of the molecular target and associated neural pathways of latrepirdine likely impeded its continued clinical development. Many possible pharmacological targets have been proposed for latrepirdine including histamine H_1_ and 5-HT_6_ receptor antagonism (Bezprozvanny, 2010), NMDA receptor modulation (Bachurin et al., 2001), improved mitochondrial function (Tempio et al., 2020), activation of AMPA kinase (Weisová et al., 2013), reduction of tau-mediated neuropathology (Peters et al., 2013) and inhibition of acetylcholinesterase activity (Giorgetti et al., 2010). Based on the literature, both the 5-HT_6_ and 5-HT_7_ receptors seem like possible candidates for relevant pharmacological targets.

In this report, we characterized latrepirdine *in vitro* against 5-HT_7_ and 5-HT_6_ receptors to determine the potency at those targets. We then used *in vivo* microdialysis to determine the doses of latrepirdine that would provide sufficient drug in brain to block those receptors. Next, we used those doses in preclinical models related to stress, agitation, anxiety and aggression with the goal of determining if these domains might contribute to the change in NPI scores noted in the clinic. The models included the elevated plus maze with a yohimbine or CCK-4 challenge, the resident intruder assay, marble burying, forced swim test and open space swimming, and sleep hypnogram. There were four major objectives for these studies: (1) Are free levels of drug detectable in rat brain after dosing? (2) If so, are the free brain levels at effective doses consistent with the affinity of latrepirdine for any pharmacological site? (3) Because many factors contribute to the NPI score, could preclinical behavioral models indicate which NPI domains might be of particular importance to the NPI score changes that were measured in patients? (4) Do the effects of latrepirdine persist after repeat dosing and are the effects reversible when dosing is stopped?

## 2 Materials and methods

### 2.1 Drugs and reagents

For both *in vitro* assays and *in vivo* experiments Latrepirdine dihydrochloride (HY-14537) was purchased from MedChemExpress, NJ, United States. Drugs used as positive controls and other standard reagents were supplied by the respective CROs.

### 2.2 *In Vitro* binding: 5-HT

#### 2.2.1 Site

Studies were conducted by Eurofins Panlabs Discovery Services Taiwan, Ltd. (New Taipei City, Taiwan).

#### 2.2.2 Procedure

CHO-K1 cells were stably transfected with a plasmid encoding the human serotonin 5-HT_7_ receptor. A 50 μg aliquot of membrane was incubated with 5.5 nM [^3^H]LSD for 120 min at 25 °C in a buffer consisting of 50 mM Tris-HCl, pH 7.4, 10 mM MgCl_2_, 0.5 mM EDTA. The K_d_ of LSD for the 5-HT_7_ site is 7.4 nM. Non-specific binding was estimated in the presence of 10 µM serotonin. Specific binding was 90%. B_max_ was 0.95 pmol/mg protein.

Competition for the LSD-labelled 5-HT_7_ site was measured in the presence of increasing concentrations of latrepirdine. The vehicle was a 1% DMSO in buffer solution. Membranes were filtered and washed 3 times and the filters are counted to determine [^3^H]LSD specifically bound.

### 2.3 Adenylyl cyclase: 5-HT_7_

#### 2.3.1 Site

Studies were conducted by Eurofins Panlabs Discovery Services Taiwan, Ltd. (New Taipei City, Taiwan).

#### 2.3.2 Procedure

CHO-K1 cells were stably transfected with a plasmid encoding the human serotonin 5-HT_7_ receptor. Test compound and/or vehicle were incubated with the cells (2 × 10^6^/mL) in HBSS buffer, 5 mM HEPES, 0.1% BSA, 100 μM IBMX, pH 7.4 and incubated at 37 °C for 20 min. The reaction was evaluated for cAMP levels by time-resolved fluorescence resonance energy transfer TR-FRET. Reaction mix was incubated with increasing concentrations of the test compound, latrepirdine, and the inhibition of 5-carboxamidotryptamine (5^−ΔΔCT^)-stimulated cAMP accumulation was determined. The vehicle was a 0.40% DMSO solution in buffer.

### 2.4 Adenylyl cyclase: 5-HT_6_

#### 2.4.1 Site

Studies were conducted by Eurofins Panlabs Discovery Services Taiwan, Ltd. (New Taipei City, Taiwan).

#### 2.4.2 Procedure

Human recombinant serotonin 5-HT_6_ receptors were stably expressed in HeLa cells. Test compound and/or vehicle were incubated with the cells (2 × 10^6^/mL) in HBSS buffer, 5 mM HEPES, 0.1% BSA, 100 μM IBMX, pH 7.4 and incubated at 37 °C for 20 min. Reaction mix was incubated with increasing concentrations of the test compound, latrepirdine, and the inhibition of 5-HT-stimulated cAMP accumulation was determined. The vehicle was a 0.40% DMSO solution in buffer. The reaction was evaluated for cAMP levels by time-resolved fluorescence resonance energy transfer TR-FRET.

### 2.5 Microdialysis and plasma exposures

#### 2.5.1 Site

Studies were conducted by Charles River Laboratories (South San Fransisco, CA). Experiments were conducted in accordance with protocols approved by the Institutional Animal Care and Use Committee of Charles River Laboratories, SSF.

#### 2.5.2 Animals

Sixty Sprague Dawley rats were group-housed in polycarbonate cages (2–3/cage) and acclimated for at least 3 days prior to commencing studies. Animals were housed in a 12 h light/dark cycle with room temperature maintained at 22 °C ± 2 °C and approximately 50% humidity and received food and water *ad libitum*. Experiments were conducted in accordance with protocols approved by the Institutional Animal Care and Use Committee of Charles River Laboratories SSF.

#### 2.5.3 Surgery

Rats pre-cannulated with a cannula in the jugular vein were utilized for the study. Rats were anesthetized using isoflurane (2%, 800 mL/min O_2_). Bupivacaine was used for local anesthesia and carprofen was used for peri-/post-operative analgesia. Animals were placed in a stereotaxic frame (Kopf instruments, United States of America). Then, MetaQuant microdialysis probes (PAN or RC; SEE Table 1) were implanted into the PFC (4 mm exposed surface). Coordinates for the tips of the probes for the PFC were: antero-posterior (AP) = +3.4 mm from bregma, lateral (L) = −0.8 mm from midline and ventral (V) = −5.0 mm from dura, the tooth bar set at −3.3 mm. After surgery, animals were housed individually in cages and provided with food and water *ad libitum*.

**TABLE 1:** Coordinates of stereotaxic surgery.

Anesthesia	Isoflurane 2%, O_2_, bupivacaine for local anesthesia, carprofen for peri/post-operative analgesia
Probes	MQ-NM- PAN or MQ-NM-RC 060–040 - PFC 4 mm exposed
Brain area	Prefrontal cortex
Coordinates	Bregma: +3.4 mm; lateral: −0.8 mm; ventral: −5.0 mm (from dura Toothbar set at −3.3 mm
Cannulation	Jugular vein

#### 2.5.4 Microdialysis

Microdialysis was performed approximately 24 h following surgery. The microdialysis probes were connected with flexible PEEK tubing to a micro-perfusion pump (Harvard PHD 2000 Syringe pump Holliston, MA). Microdialysis probes were perfused with aCSF containing 147 mM NaCl, 3.0 mM KCl, 1.2 mM CaCl_2_, 1.2 mM MgCl_2_ + 0.2% BSA, at a slow flow rate of 0.15 μL/min and a carrier flow (UP + 0.15% BSA) at a rate of 0.8 μL/min (SEE Table 2). Microdialysis samples were collected for 30-min periods by an automated fraction collector (820 Microsampler, Univentor, Malta) into 300 μL polypropylene mini-vials. After stabilization, one baseline sample was collected, and the test compound was given sublingual as described in Table 2. Then, eight additional 30-min ISF samples from the PFC were collected (for a total collection time of 5 h). All ISF samples were stored at −80 °C till analysis by CRL.

**TABLE 2:** Microdialysis details.

Prestabilization (h)	1 (including flow check)
Dialysis slow flow (µL/min)	0.15
Carrier flow (uL/min)	0.8
Ringer solution	aCSF (147 mM NaCl, 3.0 mM KCl, 1.2 mM CaCl_2_, 1.2 mM MgCl_2_)
Number of samples	9
Carrier fluid	Ultrapure water +0.2% BSA
Sample time (min)	30
Probe type	MQ-NM- PAN or MQ-NM-RC 060–040

In addition to ISF collection, blood samples were collected via the JV cannula at 0, 30, 60, 120, 180 and 240 min after dosing into EDTA anticoagulant vials (∼200 μL blood/sample) and kept on ice until processed for plasma (centrifugation at 4 °C, 2500 g for 10 min). The plasma (∼50 μL) samples were aliquoted into separate 1.5 mL Eppendorf vials and stored at −80 °C awaiting analysis by CRL.

### 2.6 Elevated plus maze (EPM) test

The EPM is used to assess the anxiety level of the test species by measuring the frequency that they enter as well as the time they spend in the open arm of the maze (Walf and Frye, 2007). Anxiety is induced in rodents with test agents like yohimbine or CCK-4.

#### 2.6.1 Site

The study was conducted by Neurofit (Illkirch-Graffenstaden, France). The experiments were conducted in compliance with animal health regulation. In particular, these regulations are: (A) with the legislation and regulations of French law (European Directive 2010/63/EU incorporated in French law, amended by Decree No. 2013–118); B) in compliance with Association for Assessment and Accreditation of Laboratory Animal Care International (AAALAC).

#### 2.6.2 Animals

Male Wistar rats (∼220 g; purchased from Janvier; Le Genest St Isle, France) were used for this study. They were group-housed (3 – 4 rats per cage) and maintained in a room with controlled temperature (21 °C–22 °C) and a reversed light-dark cycle (12 h/12 h; lights on: 17:30–05:30; lights off: 05:30–17:30) with food and water available *ad libitum*. EPM apparatus was a PVC maze covered with Plexiglas and subdivided into four equal exploratory arms (40 × 10 cm), which were all interconnected by a small platform (10 × 10 cm). The apparatus was placed 65 cm above the floor. Two arms were open, and two others were closed with transparent walls (high: 10 cm). The elevated plus maze test was done in lighting conditions of 100–150 lux light.

#### 2.6.3 Procedure

All compounds/drugs were prepared in saline (0.9% NaCl). After the administration of compounds, rats were placed on the platform opposite a closed arm in the elevated plus maze. The number of entries and the time spent in each open arm were recorded during a 5 min’ period. The animal was considered as entered in an arm when it placed its four paws in the arm.

For **Yohimbine EPM** test, yohimbine (2.5 mg/kg, i.p.) was administered intraperitoneally to all the treatment groups, except for the vehicle control group, 30 min prior to the test. Latrepirdine (0.1, 0.3, 1 and 3 mg/kg, i.m.) was administered 60 min prior to the test (30 min prior to yohimbine administration). Diazepam (1 mg/kg, i.p.) was administered 30 min prior to the test (at the time of yohimbine administration).

For **CCK-4 EPM** test, CCK-4 (0.2 mg/kg, i.p.) was administered intraperitoneally to all the treatment groups, 30 min prior to the test. Latrepirdine (1, 3, 10 and 15 mg/kg, i.m.) and diazepam (1 mg/kg, i.p.) were administered 60 min prior to the test i.e. 30 min prior to CCK-4 administration. In the pilot study, only the vehicle and CCK-4 (0.2 mg/kg) were administered to rats followed by the EPM test.

#### 2.6.4 Data analysis

Data from EPM (both Yohimbine and CCK-4) were analyzed using one-way ANOVA followed by Dunnett’s test.

### 2.7 Resident-intruder test (RIT)

#### 2.7.1 Site

Studies were conducted by Suven Life Sciences Limited (Hyderabad, India). All procedures were followed as per the guidelines provided by the Committee for Purpose of Control and Supervision of Experiments on Animals (CPCSEA; since 2023 it is CCSEA) India. The study design and procedures followed herein have been approved by the Institutional Animal Ethics Committee (IAEC) of Suven Life Sciences Limited.

#### 2.7.2 Animals

Male Swiss albino mice (10–40 g) and female Swiss albino mice, (10–30 g) were purchased from Vivo Bio Tech Ltd., Hyderabad, India. Male Swiss albino mice (10–20 g, intruder animals) were housed 5 animals/cage for a period of 13 days (social habituation). Standard laboratory conditions, such as temperature and relative humidity were maintained 21 °C ± 3 °C and 30%–70%, respectively. 12 h light/dark cycle (lights on between 07:00–19:00 h) was used. Food and water were provided *ad libitum*.

#### 2.7.3 Surgery

Bilateral ovariectomy surgery was carried for female Swiss albino mice (Alagwu and Nneli, 2005). Animals were allowed to recover for a period of 3 weeks (surgery was carried out by a single experimenter).

#### 2.7.4 Procedure

The procedure described by Koolhaas et al., 2013, was followed in this study. Male Swiss albino mice (resident animals) were housed individually with an ovariectomized female Swiss albino mouse for a period of 29 days (isolated habituation). On day 30 and 31 of the study, female mouse was removed from the resident cage, and an intruder was placed in the home cage of resident animal for a period of 10 min. During this 10 min’ exposure, the aggressive behavior (like tail rattling, chasing, biting, lateral attack, clinch attack) of the resident animal was noted as duration of attack. On day 33 of the study, latrepirdine (0.3, 1, 3 and 10 mg/kg; i.p.)/vehicle (i.p.) was administered to the resident animal 60 min prior to trial. Animals in the positive control group were administered sodium valproate (300 mg/kg, i.p.) 30 min prior to trial. After post treatment interval, same intruder was exposed to the same resident animal for a period of 10 min, and the duration of attack was noted. Each treatment group consisted of 7–14 animals.

#### 2.7.5 Data analysis

Data from RIT was analyzed using one-way ANOVA followed by Bonferroni’s *post hoc* test.

### 2.8 Open field test (OFT)

#### 2.8.1 Site

Studies were conducted by Suven Life Sciences Ltd. (Hyderabad, India). All procedures were followed as per the guidelines provided by the Committee for Purpose of Control and Supervision of Experiments on Animals (CPCSEA) India. The study design and procedures followed herein have been approved by the Institutional Animal Ethics Committee (IAEC) of Suven Life Sciences Limited.

#### 2.8.2 Animals

Swiss albino mice (30–40 g) were used for the study. Standard laboratory conditions, temperature etc. were maintained between 21 °C ± 3 °C and relative humidity between 30%–70% under a 12 h light/dark cycle (lights on between 07:00–19:00 h). Food and water were given *ad libitum*. Mice were acclimatized to the environment for at least 7 days. The open field test was done in lighting conditions of 40 lux light.

#### 2.8.3 Procedure

The spontaneous locomotor activity in terms of total distance travelled is measured in an open field test. Comparatively, lower distance travel would indicate either less motor activity or sedation. Locomotor activity was assessed using the Hamilton Kinder apparatus. On the day of testing, animals were acclimated in the experimental room for at least 1 h prior to administering vehicle or latrepirdine. Latrepirdine was dosed at 1, 3 or 10 mg/kg, intraperitoneally, 1 h prior to test. Locomotor activity was recorded for 30 min using software (Video mot 8.03, TSE systems). Each treatment group consisted of 12 animals.

#### 2.8.4 Data analysis

Data from OFT was analyzed using one-way ANOVA followed by Bonferroni’s *post hoc* test.

### 2.9 Marble burying test (MBT)

#### 2.9.1 Site

The study was conducted by Neurofit (Illkirch-Graffenstaden, France). The experiments were conducted in compliance with animal health regulation–in particular; A) with the legislation and regulations of French law (European Directive 2010/63/EU incorporated in French law, amended by Decree No. 2013–118); B) in compliance with Association for Assessment and Accreditation of Laboratory Animal Care International (AAALAC).

#### 2.9.2 Animals

CD-1 mice (5 weeks old; purchased from Janvier; Le Genest St Isle, France) were used in marble burying test.

#### 2.9.3 Procedure

The marble burying test is used to record the number of marbles buried by mice placed in a novel environment. Mice, which are placed individually in a cage, bury glass marbles that are present in the cage. This test assesses obsessive or repetitive behavior in rodents (Thomas et al., 2009; Angoa-Pérez et al., 2013).

Diazepam was prepared in normal saline (0.9% NaCl) and latrepirdine was prepared in PBS. Latrepirdine (1, 3, 10 and 30 mg/kg, i.p.) and diazepam (1 mg/kg, i.p.) were administered for 60 min and 30 min, respectively, before behavioral test. Each animal was placed individually in the cage, post treatment, without food or water, where it remained for a 20-min’ test session. On termination of the test session, the animals were removed from the cage and the number of marbles buried in the sawdust were recorded by the experimenter. Each treatment group consisted of 20 animals.

#### 2.9.4 Data analysis

Data from MBT was analyzed using one-way ANOVA followed by Dunnett’s test.

### 2.10 REM sleep

#### 2.10.1 Site

Studies were conducted by PsychoGenics Inc. (Paramus, NJ). All housing and testing of the animals were in accordance with the Principles of Laboratory Animal Care and the approval of the PsychoGenics, Inc. Institutional Animal Care and Use Committee in Association for Assessment and Accreditation of Laboratory Animal Care International (AAALAC).

#### 2.10.2 Animals

Twelve adult male Sprague-Dawley rats (∼270–300 g on arrival; source: PsychoGenics Inc., Paramus, NJ) were used for sleep study. During this study, a 12-h light/dark cycle was maintained. The room temperature was maintained between 20 °C–23 °C with a relative humidity maintained near 50%. Chow and water were provided *ad libitum* for the duration of the study.

#### 2.10.3 Procedure

Recordings started 2 h prior to latrepirdine (3 and 10 mg/kg) or vehicle administration (by intramuscular injection) and recorded continuously for 24 h thereafter. Rats were tested twice weekly in a cross over design with a minimum of 3-day washout period between doses. Electroencephalography data were read into NeuroScore™ software (Data Sciences International) for visualization, processing, and analyses. Sleep stages were assigned in 10-s epochs using electroencephalography, electromyography, and locomotor activity. Four sleep stages were defined: Active Wake; Quiet Wake; Slow Wave Sleep (SWS) or NREM sleep, and paradoxical or REM sleep. Fast Fourier Transform was used for spectral analysis. Spectral power was measured for each 10 s epoch at 1 Hz resolution (1–100 Hz) and summed to yield cumulative power.

#### 2.10.4 Data analysis

Data from REM Sleep were analyzed using one-way ANOVA followed by Dunnett’s test.

### 2.11 Forced swim test (FST)

#### 2.11.1 Site

Studies were conducted by Eurofins Advinus Ltd. (Bengaluru, India). All procedures were followed as per the guidelines provided by the Committee for Purpose of Control and Supervision of Experiments on Animals (CPCSEA; since 2023 it is CCSEA) India. The study design and procedures followed herein have been approved by the Institutional Animal Ethics Committee (IAEC) of Eurofins Advinus Limited.

#### 2.11.2 Animals

Sprague-Dawley male rats (9–10 weeks old), purchased from Hylasco Biotechnology Pvt. Ltd., India 500,078, were used for this study. Rats were housed in an environment-controlled room at 22 °C ± 3 °C and relative humidity of 30–70 percent. During the study, 12/12 light/dark cycles were maintained. Lights of the acclimatization area were switched off at 06:00 p.m. Food and water were provided *ad libitum*.

#### 2.11.3 Procedure

All experiments were carried out at ambient temperature under dark phase of lighting (between 06:00 p.m. to 06:00 a.m.). Animals were randomized based on body weights such that the inter-group variation did not exceed ±10% of the mean body weight across the groups. The test was carried out in transparent cylindrical glass containers measuring 46 cm in height and 20 cm in diameter. The containers were filled with water (23 °C–25 °C) to a depth of 30 cm.

There were 2 swimming sessions, 24 h apart. The first session (day 1) was for 15 min, called the pretest/training stage and the second session (day 2) was for 6 min, called test stage. On day 2, animals were administered with vehicle (0.1 mL, intramuscular), desipramine hydrochloride (30 mg/kg per oral) or latrepirdine (1 or 3 mg/kg, intramuscular), 1 h prior to test. Both desipramine and latrepirdine were formulated in normal saline. Scoring was done manually with no video recording. On day 2 (test stage), the first 1-min data was excluded from analysis, and the rest 5 min data was considered. During the test stage, animals were observed for floating with the absence of any movement (immobility), climbing, and swimming. The duration of time spent immobile, swimming, and climbing was observed by an observer blind to the treatment group.

Observation Criteria: a) Immobility period: A rat was judged to be immobile when it remained floating in the water without struggling and was making only those movements necessary to keep its head above water. b) Swimming behavior: A rat was judged to be swimming if it showed active horizontal (swimming) motions, more than necessary to merely maintain its head above water (e.g., moving around in the cylinder). c) Climbing behavior: A rat was judged to be climbing when it showed active vertical movements with its forepaws in and out of the water, usually directed against the walls.

#### 2.11.4 Data analysis

Data from FST were analyzed using one-way ANOVA followed by Dunnett’s test.

### 2.12 Open space swim test (OSST)

#### 2.12.1 Site

Study was conducted by Porsolt S.A.S. (Le Genest-Saint-Isle, France). The study was conducted in compliance with Animal Health regulations, in particular: A) In accordance with the Porsolt facility accreditation for experimentation; and B) In accordance with the recommendations of the Association for Assessment and Accreditation of Laboratory Animal Care (AAALAC).

#### 2.12.2 Animals

Swiss albino mice were purchased from Janvier; Le Genest St Isle, France and group housed in makrolon cages (6 animals per cage). Environmental enrichment such as gnawing and nesting material and tunnel was provided. The animal house was maintained under artificial lighting (12 h) between 7:00 and 19:00 in a controlled ambient temperature of 22 °C ± 2 °C.

#### 2.12.3 Procedure

Mice, when placed in a cylinder filled with water, become immobile. Acute antidepressants decrease the duration of immobility in normal mice. Repeated swimming in large open space increases immobility and delays the effects of antidepressants as described by Sun and Alkon (2003) in rats and adapted to mice by Stone et al. (2008).

Mice were individually placed in a makrolon cage (41 × 25 × 18 cm) containing 10 cm water (22 °C) from which they could not escape, for 15 min daily during 5 consecutive days (Day 1 to Day 5) and then, on Days 8, 10, 12, 15, 16, 19, 22, 24 and 26. All swimming sessions were video-recorded, and the behavior of animals was analyzed using a video-tracking system (Panlab: SMART). The principal measures taken were the duration of immobility, the distance travelled, and the time spent in the periphery of the arena during each trial.

Animals in group 1 were administered vehicle throughout the duration of the experiment (days 1–26). Animals in group 2 were administered vehicle during the training phase (days 1–7) and then the washout phase (days 16–22). During treatment phase, they were administered latrepirdine, 20 mg/kg from days 8–11, and 40 mg/kg from days 12–15; during reinstatement phase (days 23–26). Drug treatment and swim test schedule have been summarized in Table 3.

**TABLE 3:** Open space swim test.

Treatment	Groups	Days																									
		1	2	3	4	5	6	7	8	9	10	11	12	13	14	15	16	17	18	19	20	21	22	23	24	25	26
Vehicle	Group 1 (N=12)	Vehicle																									
Latrepirdine (LAT, mg/kg)	Group 2 (N=12)	Vehicle							20	20	20	20	40	40	40	40	Vehicle							40	40	40	40
Swim Test	OSST	Y	Y	Y	Y	Y	-	-	Y	-	Y	-	Y	-	-	Y	Y	-	-	Y	-	-	Y	-	Y	-	Y

#### 2.12.4 Data analysis

Data from OSST was analyzed by Welch’s unpaired t-test.

### 2.13 Data analysis

All the behavioral data were analyzed using GraphPad Prism® software (Version 10.2.3). Data from EPM (Yohimbine), EPM (CCK-4), FST, and MBT were analyzed using one-way ANOVA followed by Dunnett’s test. RIT and OFT data were analyzed using one-way ANOVA followed by Bonferroni’s *post hoc* test. OSST data was analyzed by Welch’s unpaired t-test. In all analyses, *p* values <0.05 were considered significant.

All the animal studies were carried out according to applicable animal ethical guidelines. All behavioral parameters were observed by observer(s) blind to the treatment group.

## 3 Results

### 3.1 *In Vitro* pharmacology

**FIGURE 1:**
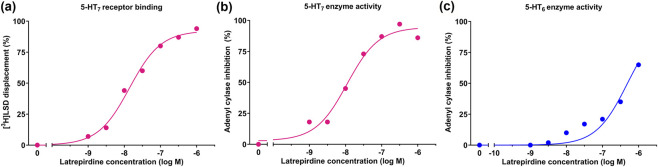
*In vitro* potency. The potency of latrepirdine for the 5-HT_7_ receptor was measured by **(a)** Displacement of [^3^H]LSD binding. **(b)** by inhibition of 100 nM 5-carboxamidotryptamine-stimulated adenylyl cyclase activity. **(c)** The potency of latrepirdine for the 5-HT_6_ receptor was measured by inhibition of 0.3 µM serotonin-stimulated adenylyl cyclase activity in cells transfected with the human 5-HT_6_ receptor. Each point represents the mean of 2 replicates. Dose-response curves were constructed using a 3-parameter logistic equation and GraphPad Prism software.

The affinity and potency of latrepirdine for the 5-HT_7_ receptor were determined using human 5-HT_7_ receptor-transfected cells and either (Figure 11) displacement of LSD binding or (Figure 1b) inhibition of 5-carboxamidotryptamine-stimulated adenylyl cyclase activity. The potency of latrepirdine was calculated as 14 nM (displacement binding) or 11 nM (enzyme activity), or approximately 4 ng/mL (the molecular weight of latrepirdine is 319). In contrast, latrepirdine is nearly 50-fold less potent at the 5-HT_6_ receptor as measured by inhibiting 5-HT-stimulated adenylyl cyclase activity in 5-HT_6_ receptor-transfected HeLa cells (Figure 1c).

### 3.2 Microdialysis

**FIGURE 2:**
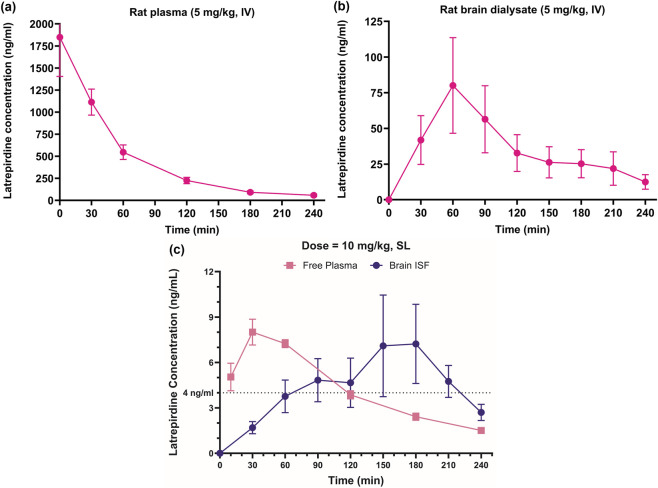
Free plasma and brain levels after dosing. Intravenous dosing. In **(a,b)**, rats were dosed at 5 mg/kg intravenously (in order to eliminate complications associated with oral bioavailability). **(a)** Plasma concentrations of latrepirdine were measured and converted to free assuming latrepirdine is 84% bound to plasma proteins. **(b)** Rat brain dialysate was obtained using microdialysis probes. Data in both **(a,b)** are presented as mean ± SEM, n = 5. **(c)** Sublingual dosing: Rats were dosed with 10 mg/kg latrepirdine sublingually. Latrepirdine was measured in plasma and brain dialysate at the indicated time points. Data are the mean and SEM of 7 measurements.

Microdialysis of free brain levels was used to determine if latrepirdine is freely permeable from plasma to brain and to calculate the doses that achieved free brain levels consistent with the affinity of latrepirdine for the 5-HT_7_ receptor (4 ng/mL). Successful CNS drugs possess the attribute of achieving high free brain concentration relative to free plasma levels after dosing (Loryan et al., 2022). In Figure 2 panels (a) and (b), rats were dosed with 5 mg/kg latrepirdine intravenously. The area under the curve (AUC, 0–4 h) was 1.77 mg*h/L for plasma and 0.15 for brain dialysate. Correcting for free plasma levels by using plasma protein binding of latrepirdine (84%), yields an AUC_plasma, u_ of 0.28. The results indicate that free latrepirdine is highly permeable with free brain levels greater than 50% of the free plasma as measured by AUC. Assuming that blood brain barrier permeability is similar across species, predictions of brain levels may be made in humans based on plasma levels in clinical studies.

We used sublingual dosing at 10 mg/kg to determine if free brain levels would be achieved necessary to block the 5-HT_7_ receptor. As shown in Figure 2c, a 10 mg/kg dose, sublingual, resulted in free brain dialysate levels above 4 ng/mL (about 12 nM) from about 1 to 3 h after dosing. These results indicate that at 1 h post dosing of 10 mg/kg sublingual, brain levels in rats should be sufficient to block the 5-HT_7_ receptor in brain. For behavior experiments we used a range of 1–10 mg/kg to test for effects of latrepirdine at central 5-HT_7_ receptors. Because equivalent doses in mice are usually double those of rats on a kilogram basis (Nair and Jacob, 2016), we used a range of 2–20 mg/kg for mouse studies.

### 3.3 Elevated plus maze test

**FIGURE 3:**
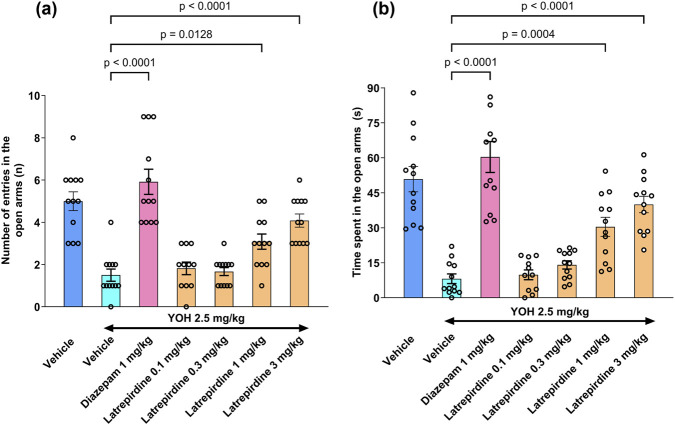
Elevated Plus Maze after Yohimbine Challenge Latrepirdine (0.1, 0.3, 1 and 3 mg/kg, i.m.) increased time spent **(a)** and number of entries **(b)** in the open arms of elevated plus maze in yohimbine (2.5 mg/kg, i.p.) administered Wistar rats. Observation time for EPM was 5 min. Data represent mean ± S.E.M., n = 12.

**FIGURE 4:**
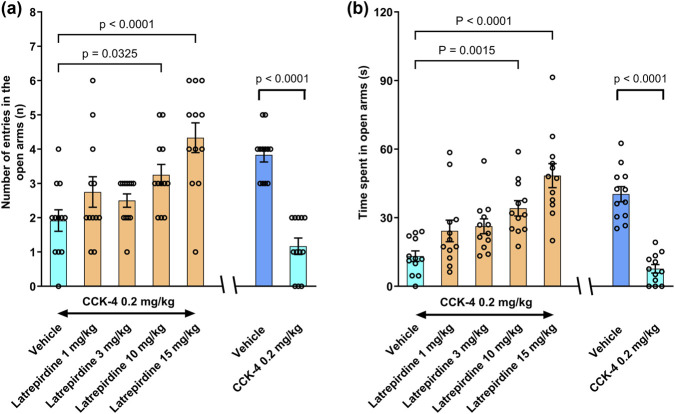
Elevated Plus Maze after CCK-4 Challenge Latrepirdine (1, 3, 10 and 15 mg/kg, i.m.) increased time spent **(a)** and number of entries **(b)** in the open arms of elevated plus maze in CCK-4 (0.2 mg/kg, i.p.) administered Wistar rats. Corresponding vehicle control are in the insets. Observation time for EPM was 5 min. Data represent mean ± S.E.M., n = 12.

Both yohimbine, an alpha_2_-adreneoceptor antagonist, and the peptide CCK-4, a cholecystokinin receptor agonist, are known to increase anxiety-like behaviors in rodents and humans (Charney et al., 1992; Rex et al., 1994; Eser et al., 2005; Zheng and Rinaman, 2013). Both yohimbine (Figure 3) and CCK-4 (Figure 4) were used to establish a deficit in the elevated plus maze (EPM), an assay used to determine efficacy of anxiolytic drugs.

Yohimbine (2.5 mg/kg, i.p.) administration significantly reduced the number of entries and the time spent in the open arms as compared to vehicle administered rats, a potential model of anxiety-like behavior. Latrepirdine (1 and 3 mg/kg, i.m.) increased number of entries and time spent in the open arms of elevated plus maze in yohimbine-treated Wistar rats as compared to yohimbine treated group, suggesting it possesses anxiolytic effects (Figure 3) [Number of entries in the open arms: F_(5, 65)_ = 22.92, *p* < 0.0001; Time spent in the open arms: F_(5, 65)_ = 29.19, *p* < 0.0001]. Diazepam (1 mg/kg), positive control, caused a significant increase in the number of entries and the time spent in open arms in the elevated plus maze when compared with the yohimbine-treated group.

CCK-4 is a synthetic analog of the endogenous neuropeptide cholecystokinin (CCK). In rats, administration of CCK-4 (0.2 mg/kg, i.p.) results in a decrease in number of entries and time spent in open arms in the elevated plus maze compared to vehicle-treated rats (Figures 4a,b, right inset). The number of entries and the time spent into the open arms were 3.8 ± 0.2 and 40.3 ± 3.3 s, respectively for the vehicle treated rats. In vehicle-administered CCK-4 treated rats, these parameters were reduced to 1.2 ± 0.2 and 7.7 ± 1.9 s, respectively [Unpaired t-test–Number of entries in the open arms: *p* < 0.0001; Time spent in the open arms: *p* < 0.0001]. Latrepirdine treatment nearly reversed the deficit induced by CCK-4 at 10 mg/kg and completely at 15 mg/kg doses (Figures 4a,b) [Number of entries in the open arms: F_(4, 55)_ = 6.744, *p* = 0.0002; Time spent in the open arms: F_(4, 55)_ = 11.06; *p* < 0.0001].

These results indicate that doses of 3–15 mg/kg are effective in 2 models of anxiety, the yohimbine and the CCK-4 models. These doses, based on extrapolating from the microdialysis results, should be sufficient to antagonize central 5-HT_7_ receptors. Because these are gain of function tests (increase in arm entries), it is unlikely that any non-specific effects on locomotor activity or sedation would account for the results.

### 3.4 Resident-intruder test

**FIGURE 5:**
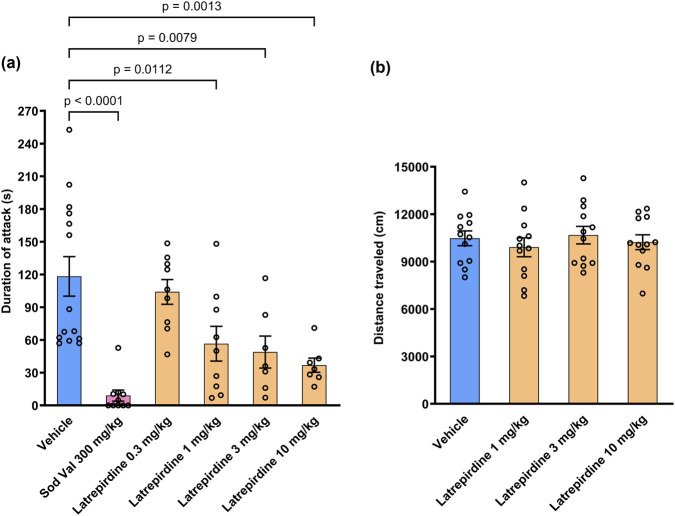
Resident Intruder and Open Field Test. **(a)** Effects of latrepirdine (0.3, 1, 3 and 10 mg/kg, i.p.) on aggression (duration of attack) in male Swiss mice. Mice were observed for a 10 minute observation period after introduction of an intruder mouse to the cage. During this 10 min’ exposure, the aggressive behavior (like tail rattling, chasing, biting, lateral attack, clinch attack) of resident animal was noted as duration of attack. Data presented as mean ± S.E.M., n = 7–14. **(b)** Mice from the resident intruder task were tested in the open field following evaluation for aggression. Latrepirdine (1, 3 and 10 mg/kg, i.p.) did not reduce spontaneous locomotor activity in male Swiss mice. Observation time for open field test was 30 min. Data presented as mean ± S.E.M., n = 12.

The resident intruder task is used to assess aggression in mice by introducing an intruder mouse into the home cage of a mouse that has been previously acclimated to the cage. The intruder mouse is introduced into the cage for 10 min. Latrepirdine dose-dependently decreased the duration of attack during the 10 min of exposure to the intruder mouse compared with the vehicle treated group. The effect was significant at doses 1 mg/kg (56.6 ± 16 s, 48%, *p* = 0.0112), 3 mg/kg (48.9 ± 14.7, 41%, *p* = 0.0079), and 10 mg/kg (36.9 ± 6.6, 31%, *p* = 0.0013, Figure 5a) (% values stated here were versus the vehicle control). The positive control (300 mg/kg sodium valproate) significantly decreased the duration of attack (9.1 ± 5.1 s, 8%, *p* < 0.0001) when compared with the vehicle treated group (118.3 ± 18.1 s) [Duration of Attack: F_(5, 50)_ = 9.185, *p* < 0.0001].

### 3.5 Open field test

To ensure that effects measured in the resident intruder test were not due to locomotor activity, the mice from the resident intruder task were allowed to recover for 3 weeks and observed in the open field test. Latrepirdine did not alter locomotor activity at any of the doses tested when compared with the vehicle treated group (Figure 5b) [Distance travelled: F_(3, 44)_ = 0.3967, *p* = 0.756]. Therefore, it is considered that effects observed in the resident intruder task were not due to any indirect effect on locomotor activity and likely due to an effect on aggression itself.

### 3.6 Marble burying test

**FIGURE 6:**
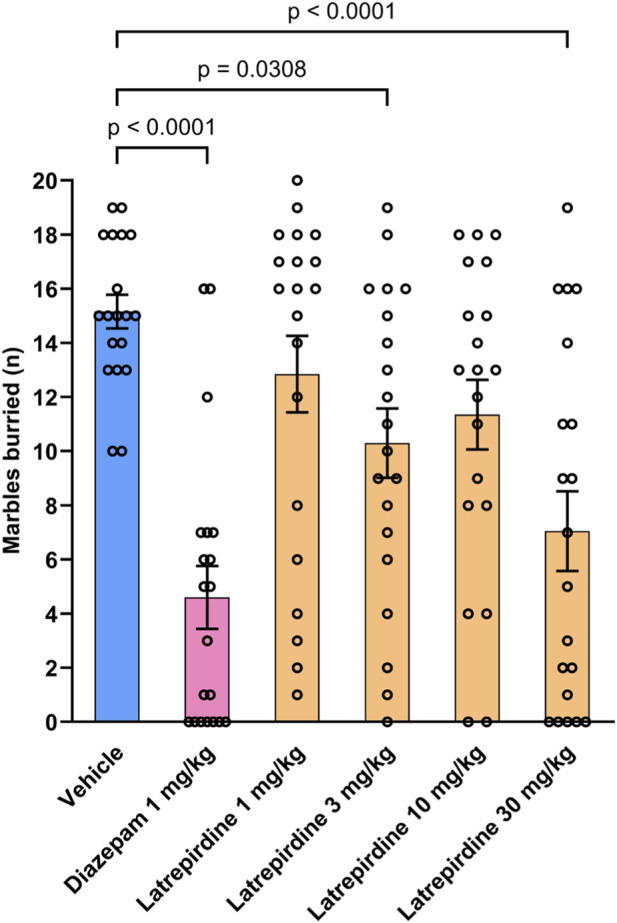
Marble Burying Latrepirdine (1, 3, 10 and 30 mg/kg, i.p.) and positive control diazepam (1 mg/kg, i.p.) decreased the number of marbles buried spontaneously by CD-1 mice. Observation time for marble burying test was 20 min and total 20 marbles were used. Data presented as mean ± S.E.M., n = 20.

The marble burying task assesses compulsive behaviors related to anxiety, obsessive compulsive disorder and stress-related psychiatric symptoms (Angoa-Pérez et al., 2013). Latrepirdine decreased the number of marbles buried when compared with the vehicle treated group. The effect was significant at doses, 3 mg/kg (10.3 ± 1.3, 68%, *p* = 0.0308), and 30 mg/kg (7.1 ± 1.5, 47%, *p <* 0.0001, Figure 6) (% values given here are relative to vehicle control). Diazepam (1 mg/kg), positive control, significantly decreased the number of marbles buried (4.6 ± 1.2, 30%, *p* < 0.0001) when compared with the vehicle treated group (15.2 ± 0.6) [Number of marbles buried: F_(5, 113)_ = 9.47, *p* < 0.0001].

### 3.7 Sleep stages study

**FIGURE 7:**
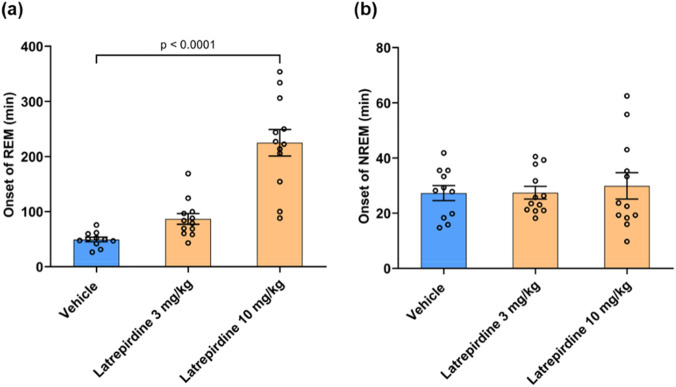
REM Sleep Effects of latrepirdine (3 and 10 mg/kg, i.p.) on **(a)** onset of REM, and **(b)** onset of NREM in minutes. Data presented as mean ± S.E.M., n = 11–12.

Latrepirdine (10 mg/kg) significantly increased the time to onset of rapid eye movement (REM) sleep (225.0 ± 24.0, 456%, *p <* 0.0001) when compared with the vehicle treated group (49.3 ± 4.1) (approximately 4 to 5-fold, Figure 7). However, latrepirdine did not show any effect on non-REM (NREM) sleep at any of the doses tested [Onset of REM: F_(2,32)_ = 40.64, *p* < 0.0001; Onset of NREM: F_(2, 32)_ = 0.1813, *p* = 0.835].

### 3.8 Stress-Induced Immobility

**FIGURE 8:**
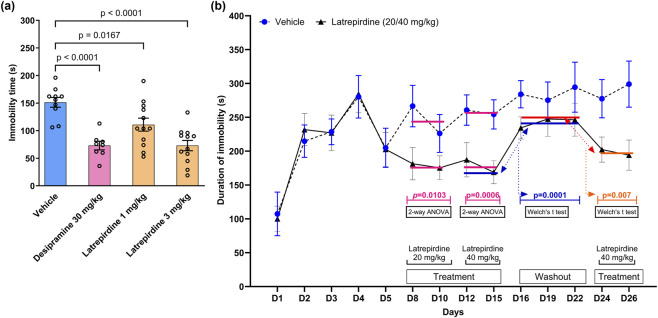
Effects of latrepirdine on stress-induced immobility: Acute and repeated dose. **(a)** Rats (SD) were dosed with a single acute dose of vehicle, latrepiridne at 1 or 3 mg/kg intramuscular, or the positive control desipramine (30 mg/kg, p.o.) 60 min before testing and then placed into a cylinder of water. Immobility time was measured in seconds during a 5 min period. Data were analyzed using one-way ANOVA followed by Dunnett’s multiple comparisons test and represented as mean ± SEM (N = 9–12 per group). In **(b)**, Swiss mice were placed into a rectangular tank filled with 10 cm of water for 5 successive days. On day 8, the mice were dosed with vehicle or latrepirdine (20 or 40 mg/kg, i.p.). Dosing was repeated on the days indicated in the figure. On washout days all animals were dosed with vehicle. All animals were dosed 1 h before testing. Immobility time was recorded during a 15 min session.

Rodents become more immobile when exposed to a stressful event. The forced swim test exposes rats to a single acute stress by placing them into a cylinder of water. A single acute dose of latrepirdine reduced immobility time (Figure 8a) and the effect was significant at doses of 1 mg/kg (111.0 ± 11.8 s, 73%, *p* = 0.0167) and 3 mg/kg (73.0 ± 9.4 s, 48%, *p <* 0.0001) using one-way ANOVA followed by Dunnett’s test. Desipramine (30 mg/kg), positive control, significantly decreased immobility time (73.0 ± 7.8 s, 48%, *p* < 0.0001) when compared with the vehicle treated group (151.4 ± 8.8 s) [Immobility time: F_(3, 38)_ = 13.27, *p* < 0.0001]. Because this is a gain of function test (increase in swim time), it is unlikely that any non-specific effects on locomotor activity or sedation would account for the results.

The open space swim task offers advantages over the forced swim test because it allows both repeated stress (placement into the tank of water on multiple days) and repeated administration of drug. Effects of repeated stress on immobility time were assessed using the open space swimming test in Swiss mice*.* Mice were randomly assigned to two groups, i.e., group 1 and group 2. In the first phase, days 1–5, all mice were placed into the water-filled tank and only vehicle was administered (Table 3). As shown in Figure 8b, all mice increased their immobility time with repeat placement into the water tank indicating poor adaptation to stress. There was no difference between groups 1 and 2 [Immobility time: F_(1, 110)_ = 0.015; *p* = 0.90]. During the 20 mg/kg treatment phase, days 8–11, there was a highly significant difference between vehicle and the 20 mg/kg dosed groups [Immobility time: F_(1, 44)_ = 7.101; *p* = 0.0107] On days 12–15 the dose of latrepirdine was increased to 40 mg/kg to determine if the 20 mg/kg dose was achieving the maximal effect. No added effect was observed after increasing the dose. During washout, days 16–22, there was a significant increase in immobility time compared to the previous treatment phase, days 8–15 (Unpaired t-test with Welch’s correction, *p* = 0.0001). This indicates that the effects of latrepirdine were fully reversible. Importantly, there was no evidence of withdrawal because the immobility time did not exceed the vehicle control. During reinstatement of 40 mg/kg dosing to group 2, from days 23–26, there was a significant decrease in immobility time compared to the washout, (from days 16–22) showing that reinstatement of latrepirdine dosing was successful (Unpaired t-test with Welch’s correction, *p* = 0.007).

## 4 Discussion

The precise molecular targets and pathways that are engaged by latrepirdine which have, thus far, remained elusive. In this study, the free brain levels of the drug at an efficacious dose were measured and compared to the potency of latrepirdine at possible targets. A 10 mg/kg dose of latrepirdine in rats achieved free brain levels of 4 ng/mL at 1-h post-dose (Figure 2c) matching the potency of latrepirdine for the 5-HT_7_ receptor, IC_50_ of 11–14 nM, or approximately 4 ng/mL (Figure 1). In the clinic, an 8.16 mg dose achieved plasma levels of 1.3 ng/mL (Chew et al., 2016). Extrapolating to a 20 mg dose, the dose that generated a possible efficacy signal in patients using the NPI (Chau et al., 2015), yields a plasma exposure of 3.2 ng/mL. Based on the free brain to plasma ratio of 50% calculated in rats (Figure 2c), we predict that human brain levels would be about 1.6 ng/mL, close to the 4 ng/mL necessary to block central 5-HT_7_ receptors. Therefore, there is consistency with respect to free brain levels at effective doses in rodents, affinity for 5-HT_7_ receptors, and estimated free bran levels in clinical studies that produced a signal in the clinical endpoint scale.

Latrepirdine generated a signal in the NPI scale across 3 clinical studies, but patient-level data is not available to determine which factors measured by the NPI contributed to the signal. The NPI measures frequency and severity of 10 items: delusions, hallucinations, dysphoria, anxiety, euphoria, aggression, apathy, irritability, disinhibition, and aberrant motor behavior (Cummings, 2020). Because 5-HT_7_ receptor antagonism likely contributes to the NPI signal observed in the clinic, we can get some clues as to the domains of the NPI that might be affected by latrepirdine by examining the preclinical and clinical effects of other 5-HT_7_ receptor antagonists. Preclinical studies with drugs with high affinity for 5-HT_7_ receptors indicates that these drugs may exhibit effects consistent with stress -induced aggression, anxiety and irritability (Mnie-Filali et al., 2011; Canale et al., 2016; Kucwaj-Brysz et al., 2018). Clinically, antipsychotic drugs such as brexpiprazole, pimozide and lurasidone have high affinity for 5-HT_7_ receptors and this may contribute to their therapeutic profile (Fukuyama et al., 2023). Brexpiprazole was recently approved for treatment of agitation (Behl et al.,2024), pimozide is prescribed for compulsive behaviors such as Tourette’s Syndrome (Pringsheim and Marras, 2009), and lurasidone has shown superiority over other antipsychotics in reducing anxiety (Goldberg et al., 2023). Based on these preclinical and clinical reports, latrepirdine may have produced a signal in the NPI by affecting domains related to anxiety, agitation, compulsive behaviors (aberrant motor activity) and aggression.

Animal assays that are used to model anxiety, aggression and compulsive behaviors include the elevated plus maze, resident intruder test, marble burying and forced swim test. Latrepirdine was effective in reversing yohimbine-induced deficits in the elevated plus maze in rats (Figure 3). Because yohimbine activates the locus coeruleus, the main source of norepinephrine-containing projections to the cortex, latrepirdine may be able to attenuate excessive noradrenergic signaling that contributes to anxiety and aggression. Similarly, latrepirdine was effective in reducing CCK4-induced deficits in the elevated plus maze (Figure 4), a useful translational model for panic and anxiety. Anxiolytics such as benzodiazepines apparently reduce CCK-4 mediated panic in healthy volunteers (Zwanzger et al., 2003).

To measure aggressive behaviors, rats were evaluated in the resident intruder test. Our results indicate that latrepirdine is highly effective in reducing aggressive behaviors associated with introduction of a resident intruder into the home environment (Figure 5). Marble burying is a task related to compulsive behaviors (Angoa-Perez et al., 2013) and has also been linked to 5-HT_7_ receptor function because mice with an inactivated 5-HT_7_ receptor gene exhibit a reduction in compulsive behaviors (Hedlund and Sutcliffe, 2007). In this study, latrepirdine was effective in reducing marble burying (Figure 6). The results noted in the elevated plus maze have high predictive validity as evidenced by the effects of similar doses in the resident intruder and marble burying tasks, both of which are thought to activate the locus coeruleus (Reyes et al., 2019; Lustberg et al., 2020). Considered as a major stress response system, locus coeruleus-mediated increases in norepinephrine is critical in maintaining arousal in response to acute stress (Myers et al., 2017).

The experimental drug JNJ-18038683 is a selective 5-HT_7_ receptor antagonist with an apparent affinity of 10 nM for the 5-HT_7_ receptor. JNJ-18038683 increased latency to REM sleep in rats at 10 mg/kg, p.o. (Bonaventure et al., 2012), nearly identical to the findings with latrepirdine (Figure 7). The similarity of the effects of latrepirdine and JNJ-18038683 further supports the hypothesis that latrepirdine is achieving its biological effects by blocking 5-HT_7_ receptors. Increased REM sleep latency is a common characteristic of many neuropsychiatric drugs (Riemann et al., 2020).

Receptor profiling data was provided by Giorgetti et al., 2010 (referenced in the paper). In that paper, a broad screen was conducted of over 100 receptor, enzyme, ion channel and transporter drug targets. Only 2 targets exhibited affinities of less than 10 nM, the histamine H1 receptor and the 5-HT_7_ receptor. There is no evidence in the literature that histamine H1 receptors mediate effects on either yohimbine- or CCK-induced deficit in stress-related models. Moreover, a selective H1 receptor antagonist increased open arm entries in the elevated plus maze, (Serafim et al., 2013). Taken together, evidence suggests that the effects observed in the EPM model are likely mediated by 5-HT_7_ receptors rather than histamine H1 receptors. Latrepirdine appears not to possess sufficient potency for any other of the listed targets to account for effects in the behavioral assays reported in this study.

**FIGURE 9:**
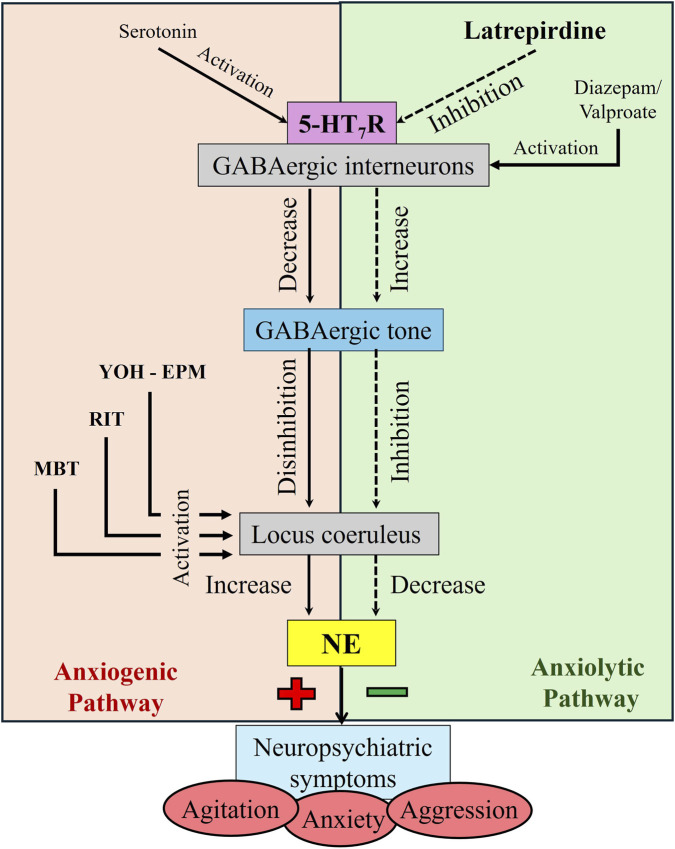
Visual Abstract. 5-HT_7_ receptors are expressed on GABAergic interneurons in the locus coeruleus and other brain nuclei. These receptors may inhibit GABAergic tone. 5-HT_7_ antagonist like latrepirdine may block 5-HT_7_ and therefore elevate GABAergic tone in the LC, reducing arousal.

Although 5-HT_7_ receptor expression in the locus coerulus has not been reported in the literature, the human protein database reports expression of 5-HT_7_ receptors in the locus coeruleus using *post mortem* tissue (The Human Protein Atlas^1^) and a high expression of 5-HT_7_ receptors in inhibitory neurons (The Human Protein Atlas^2^). Monti and Jantos (2014) conducted a study in which they microinjected a 5-HT_7_ receptor agonist LP-211 and an antagonist SB-269970 into the locus coeruleus and concluded that the 5-HT_7_ receptors might be expressed on GABAergic neurons in that region. A similar proposal has been made for the role of 5-HT_7_ receptors in the raphe (Roberts et al., 2004; Mnie-Filali et al., 2011; Kusek et al., 2015). We propose that 5-HT, by activating 5-HT_7_ receptors expressed on GABAergic neurons, inhibit these neurons (Figure 9). Antagonists like latrepirdine relieve this inhibition and decrease activity of the norepinephrine-containing principal neurons. This model would explain the effects of a 5-HT_7_ antagonist in the elevated plus maze after a yohimbine challenge. Yohimbine, by blocking auto-inhibitory alpha_2_-adrenergic receptors on the locus coeruleus neurons, would increase the output of these neurons. A 5-HT_7_ receptor antagonist would diminish the effect of yohimbine by increasing the activity of GABAergic interneurons (see the visual abstract, Figure 9). Interestingly, CCK is also expressed in inhibitory neurons in the LC and the CCK receptor is expressed on LC neurons (Barde et al., 2024). CCK receptor antagonists may reduce panic clinically (Goettel et al., 2023). This suggests that both the 5-HT_7_ receptor and CCK systems work similarly to modulate LC activity and affect panic and anxiety. Of note is that the positive control used for the resident intruder task, valproate, may also work by activating inhibitory GABAergic neurons in the locus coeruleus (Olpe et al., 1988) and is used to treat aggression clinically (Hollander et al., 2003). In summary, 4 classes of drugs that treat anxiety and aggression, including benzodiazepines (diazepam), valproate, CCK-4 receptor blockers and 5-HT_7_ receptor antagonists may all work by reducing the output of locus coeruleus neurons. This model may also explain the clinical phenomena called the Serotonin Syndrome (Simon et al., 2025). Serotonin syndrome results from excessive serotonergic signaling which, if our model is correct, would result in excessive inhibition of GABAergic interneurons leading to a hyper-excitable state. A prediction of our model is that latrepirdine might be especially useful in reducing stress-mediated behaviors under conditions in which excessive serotonergic signaling may be present.

The EPM, resident intruder, REM latency, FST and marble burying models assess properties of latrepirdine after a single acute dose. To be useful as a chronic drug for treatment of psychiatric symptoms, latrepirdine would have to be effective after repeated doses and after multiple episodes of stress. The open space swim test provides a suitable model to assess these properties of latrepirdine. In the open space swim test, repeated placement of mice into the swim chamber over 4 days markedly increases immobility time, indicating that repeated exposure to stress has a greater impact on behavior than a single acute exposure (Figure 8). Despite the greater effects of repeated exposure to stress, latrepirdine continues to be effective in reducing immobility time after multiple doses. When dosing with latrepirdine is stopped, immobility time increases to the same level as vehicle-dosed mice. When latrepirdine dosing is reinstated, immobility time again is reduced. These findings indicate that latrepirdine possesses favorable qualities for chronic administration including lack of desensitization, no withdrawal effect after stopping dosing, and reversibility after stopping and reinstating dosing. In addition to the preclinical data reported in this study, the potential to use latrepirdine as a chronic treatment is supported by the safety profile that was achieved in a patient population for up to 1 year (in this case, Alzheimer’s patients) (Chau et al., 2015).

In conclusion, latrepirdine appears to ameliorate stress-related behavioral effects in rodents by blocking the 5-HT_7_ receptor. Because 5-HT_7_ receptors are apparently expressed on GABAergic interneurons in the LC, latrepirdine may elicit these behavioral effects by blocking these 5-HT_7_ receptors and thereby increasing inhibitory tone. The positive effects on anxiety, aggressive and compulsive behaviors may correspond to the neuropsychiatric effects that have been reported in clinical studies. Lastly, latrepirdine demonstrates properties such as lack of desensitization and reversibility, in a repeat dose paradigm that suggests it would be useful for chronic dosing. Latrepirdine may be a useful drug for the chronic treatment of stress-induced and anxiety-related symptoms.

## Data Availability

The raw data supporting the conclusions of this article will be made available by the authors, without undue reservation.

## Footnotes

1 The Human Protein Atlas https://www.proteinatlas.org/ENSG00000148680-HTR7/brain/pons [accessed on 24 November 2025].

2 The Human Protein Atlas https://www.proteinatlas.org/ENSG00000148680-HTR7/single+cell [accessed on 24 November 2025].
